# A Systematic Review on Mobile Health Applications’ Education Program for Patients Taking Oral Anticoagulants

**DOI:** 10.3390/ijerph18178902

**Published:** 2021-08-24

**Authors:** Insil Jang

**Affiliations:** Department of Nursing, Chung-Ang University, Seoul 06974, Korea; shili79@cau.ac.kr; Tel.: +82-2-820-5744

**Keywords:** systematic review, oral anticoagulants, nursing, application, self-management

## Abstract

Warfarin is widely used as an oral anticoagulant. However, it is difficult to manage patients due to its narrow therapeutic range and individualized differences. Using controlled trials and real-world observational studies, this systematic review aimed to analyze health education’s impact among patients on warfarin therapy by mobile application. Smartphone and tablet applications have the potential to actively educate patients by providing them with timely information through push notifications. MEDLINE, EMBASE, CINAHL, Web of Science, and Cochrane electronic databases were searched using the keywords “anticoagulants,” “warfarin”, “mobile application”, and “smartphone” up to May 2020. Of the 414 articles obtained, 12 articles met the inclusion criteria for this review. The education and self-management programs using the mobile health application had diverse contents. A meta-analysis was not deemed appropriate because of the heterogeneity of populations, interventions, and outcomes. Thus, a narrative synthesis is presented instead. This review demonstrates that educating patients for anticoagulation management through their smartphones or tablets improves their knowledge levels, medication or treatment adherence, satisfaction, and clinical outcomes. Moreover, it has a positive effect on continuing health care. Future research concerning patients taking warfarin should include key self-management outcomes in larger, more rigorously designed studies, allowing for comparisons across studies. This study proposes a continuous application of timely education through smartphone applications to the current medical and nursing practice.

## 1. Introduction

Warfarin remains among the most commonly indicated forms of oral anticoagulant therapy for the prevention and treatment of thromboembolic complications associated with various cardiovascular diseases. Warfarin intake requires special care because it has a narrow therapeutic concentration range. Moreover, its therapeutic effect will be affected if an improper dosage is given [1]. Warfarin’s efficacy and safety require anticoagulation control’s optimal quality. This is demonstrated by a “time in therapeutic range” (TTR) of more than 70% while minimizing the risk of serious adverse bleeding events [2,3]. Therefore, oral anticoagulation therapy with vitamin K antagonists must be regularly monitored and adjusted. This is important to maintain patients’ international normalized ratio (INR) levels within the target range [4]. Additionally, food and drug interactions, alcohol consumption, other diseases, and stress may also influence INR values when taking warfarin. As such, medication adherence and continuous management are important [5]. Warfarin-related self-management is an essential factor in controlling serious complications such as heart failure, valve failure, increased medical costs, reoperation risk, poor quality of life (QOL), and death despite ongoing stress. 

Patient education is a known predictor of increased engagement in shared decision-making, improved medication and treatment adherence, higher levels of satisfaction, and even better treatment outcomes [6,7]. Medication adherence pertains to the degree to which a person’s behavior, represented by drug consumption, diet tracking, and lifestyle changes agrees with a doctor’s or other health professional’s recommendations [8]. High levels of drug knowledge and self-efficacy increase drug use level and self-care. Patients’ knowledge is an essential component in warfarin therapy for improving patient-reported outcomes (PROs) such as satisfaction and adherence [6,7,9]. Therefore, patient education is a crucial element concerning health care for patients taking oral anticoagulants.

Warfarin-controlled self-management generally encompasses a range of activities, such as tracking symptoms, increasing physical activity, adhering to a special diet, and supporting mental health, aimed at engaging patients to take an active role in their own care [10]. New approaches for chronic disease management are patient-centered, whereby the patient practices shared treatment decision-making, leading to the health system’s improved outcomes and efficacy [11,12]. At present, various smartphone applications are continuously being developed for health management. Smartphone and tablet applications have the potential to actively educate patients by providing them with timely information through push notifications. Mobile health (mHealth) technology seems promising in expanding healthcare coverage, facilitating the decision-making process, and improving chronic disease management [9,13,14]. The mHealth applications are an innovative potential solution for supporting self-management by monitoring symptoms, adhering to medication, increasing knowledge, and measuring physiological values [9,14]. These mHealth applications are used independently or under healthcare professionals’ guidance. They have evolved in terms of improving clinical outcomes among patients on warfarin therapy [15]. 

However, despite their potential, mHealth applications often lack the necessary components of self-management required to support patients with long-term conditions, such as customized medical devices [16]. The quality required by the target population used must be guaranteed for mHealth applications to function properly as a medical device. Moreover, ethical issues must be addressed, and the safety of patients’ personal data must be ensured. However, the lack of theoretical underpinning to inform and guide behavioral changes (i.e., increasing medication adherence or physical activity levels) raises questions about the applications’ benefits for their users [17,18]. At present, it is very important to understand which mHealth applications’ components affect easy adaptation and behavioral change among patients. It is likewise vital to improve knowledge and medication adherence to patients taking warfarin. Systematic reviews with content analyses have examined the quality, functionality, and clinical outcomes of mHealth apps used for hypertension, pain, and other chronic diseases [17,18,19]. Therefore, this systematic review aimed to analyze health education’s impact on patients on warfarin therapy using mobile health applications. Specifically, this review explored controlled trials and real-world observational studies. Within this review, we focused on patients that receive their care in a hospital setting. This study evaluated the elements and effectiveness of interventions using mHealth applications on outcomes such as patient knowledge of his or her disease and treatment, treatment guidelines and drug use adherence, prothrombin time levels control, and satisfaction with treatment received.

## 2. Methods

### 2.1. Literature Search Strategies

This systematic review aimed to identify the elements and effectiveness of interventions with mHealth applications on the clinical outcomes among patients taking warfarin. This study was completed using the Preferred Reporting Items for Systematic Reviews and Meta-Analyses (PRISMA) Statement [20]. In collaboration with university librarians, we conducted a comprehensive search of studies published up to May 2020. We used electronic databases obtained from MEDLINE, Embase, CINAHL, Web of Science, and Cochrane. Searches included combinations of free text words and index terms using Boolean operators such as “anticoagulants”, “warfarin”, “mobile applications”, and “smartphones” (Table 1). 

This review included studies that adhered to the following criteria: (a) had participants who were adults (18 years of age or older) with atrial fibrillation or valvular heart disease diagnoses and are currently taking warfarin, (b) had participants visiting outpatient clinics, (c) studies confirming the effects related to self-management through mobile health applications, (d) published in English or Korean, and (e) published in peer-reviewed journals and are offered in full text. 

### 2.2. Quality Assessment 

The studies were evaluated for adherence to the inclusion criteria using the Downs and Black checklist (1998). Such a checklist is suitable for assessing the quality of randomized and non-randomized studies in healthcare interventions [21]. It consists of 27 questions evaluating five subscales: research reporting, external validity, internal validity-bias, internal validity-confounding, and power [21]. One question assessing the study’s power (question 27) was modified so that it can be answered with a “yes” or “no” [22]. For question 5, a question about confounders, 0 = no, 1 = partially, and 2 = yes, for a total of 28 points. Previous literature’s criteria were applied to evaluate each study’s quality. The criteria were as follows: excellent (28–26), good (25–20), fair (19–15), and poor (≤14) [23,24]. Two reviewers (J.L. and I.J.) independently assessed 12 studies’ methodological quality in the final inclusion criteria. Their disagreements were resolved through a discussion until a consensus was reached.

### 2.3. Data Extraction

**Figure 1:**
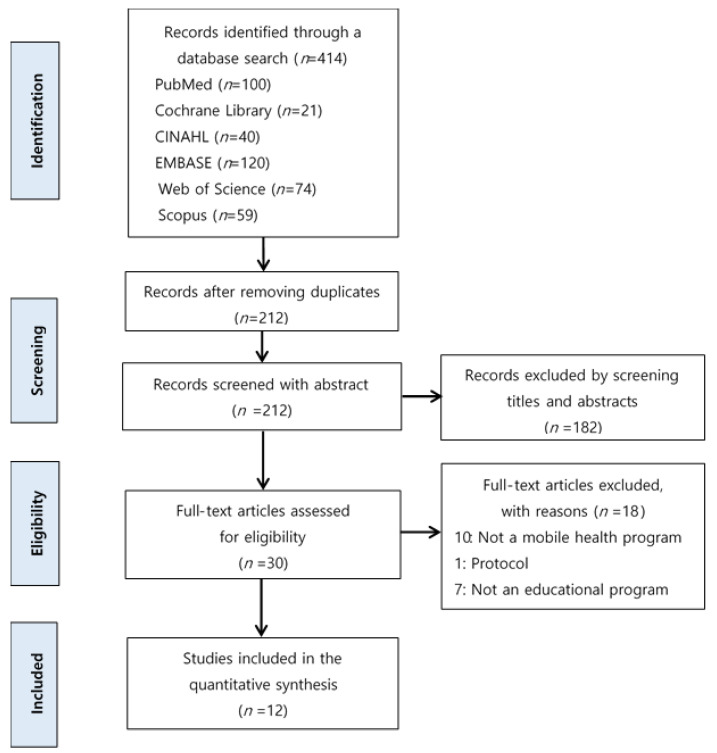
Preferred Reporting Items for Systematic Reviews and Meta-Analyses (PRISMA) flow diagram of the literature search.

The two reviewers (J.L. and I.J.) independently extracted the data for identifying potentially relevant studies. All duplicates of articles initially retrieved were removed. They then applied the inclusion and exclusion criteria by reviewing the title and abstract. They reviewed the full text when they found it difficult to accurately judge the title and abstract. Among the 212 articles with removed duplicates, 182 articles were excluded based on the title and abstract. The reviewers conducted a full-text review on a total of 30 studies. Figure 1 provides the reasons for some of the full-text articles’ exclusion. 

Data were extracted through a process using the matrix method [25]. The matrix method’s steps are as follows: (1) creating a paper trail of the search process and results, (2) organizing journal articles and other materials collected for review, (3) abstracting data from each journal article, (4) synthesizing the abstracted information, and (5) writing a literature review [25]. The reviewers abstracted and tabulated information, such as author names, year of publication, country, study design, inclusion criteria, sample characteristics, program content, intervention duration, and study outcomes. All processes were independently reviewed and agreed upon by the two reviewers (J.L. and I.J.). A third reviewer (J.K) was involved in the absence of consensus.

## 3. Results

### 3.1. Search Outcome

Figure 1 presents the study selection flow’s details. Eleven papers were selected according to the data inclusion criteria, and the selection process was employed. The literature search using each database rendered a total of 414 studies (PubMed = 100; Cochrane Library = 21; CINAHL = 40; EMBASE = 120; Web of Science = 74; and Scopus = 59). Among these studies, only 212 studies remained after eliminating duplicate articles. Furthermore, 30 articles were identified through the examination of their titles and abstracts that were potentially relevant to the intervention with mHealth applications for control warfarin. Eventually, 12 studies were selected after thoroughly assessing the article’s full texts and eliminating the studies that did not meet the selection criteria.

### 3.2. Characteristics of the Included Studies

Table 2 provides an overview of the study characteristics of the 12 studies included in this review. Five of the twelve studies were randomized controlled trials. The studies were conducted in the United States of America (*n* = 3), China (*n* = 3), Germany (*n* = 2), the Netherlands, Brazil, Taiwan, and Saudi Arabia (*n* = 1). The twelve papers to be analyzed were published in 2014, 2015, 2016, 2017 (four papers), 2018, 2019, and 2020 (three papers). More than half of the studies have been published within the last five years. In the case of randomized or non-randomized control test studies, the sample size varied from 21 to 3324. Conversely, the sample size was 195 to 7578 in the cohort study. All studies were centered on patients in hospital settings. Moreover, the study participants’ most common mean age range was in the 60s (five papers). This is followed by the 70s (three papers), 50s (three papers), and 40s (one paper). All study participants were restricted to adults taking warfarin continuously for atrial fibrillation or other reasons. 

### 3.3. Overview of the Intervention

The reported mHealth interventions in the 12 studies were markedly variable (Table 2). A cohort study was conducted with an intervention and control design except for the three studies with a one-group pre-post design. Since the program is being applied to subjects taking warfarin, the mHealth applications were named as CATA, Poltavita, Kardia, MASS, Mobile AF, and mAFA. The duration of intervention in each study varied from one month to 14 months or at every outpatient visit. Regarding the intervention method, most of the seven studies used mobile health applications on their own smartphones. The rest of the studies used a web-based platform.

The contents of the program applied in each study were very diverse. The educational program consisted of knowledge about diseases and medication, risks and benefits understanding, self-testing skills, and safety tips concerning warfarin therapies. It focused on self-care and self-management by inducing an improvement in the warfarin-taking subjects’ medication adherence and patient activation. Some programs included self-management to participate in clinical decision-making, monitoring (including INR values, symptom identification, medication dose determination algorithm), and participation in warfarin dose determination. 

#### 3.3.1. Clinical Outcomes

Six studies of clinical outcomes (i.e., PT values, TTR of INR, symptoms, complications such as bleeding, readmission, and mortality) were recorded among the 12 studies (Table 2). Prochaska et al., Lin et al., and Guo et al. only evaluated clinical outcomes without self-management outcomes [26,27,28,29]. 

#### 3.3.2. Self-Management Outcomes

Various self-management outcomes measured oral anticoagulant knowledge, QOL, efficacy, and medication adherence in a self-report format. These were reported in eight studies (Table 2). Additionally, satisfaction, usability, and feasibility were measured as the effect of intervention through mobile health apps [30,31,32,33].

As shown in Table 2, knowledge was measured as self-management outcomes in four studies. In contrast, the Oral Anticoagulation Knowledge (OAK) tool was used in two studies [30,32]. Two studies confirmed efficacy as an outcome, and the assessment tools were different [26,34]. Three papers reported QOL (i.e., HRQoL with the Atrial Fibrillation Effect on Quality of Life (AFEQT), and Patient Health Questionnaire (PHQ-9)) [33,35]. Moreover, four papers reported medication adherence (i.e., Morisky Medication Adherence Scale (MMAS)) papers [29,31,32,33]. The studies included in this paper used various assessment tools for the self-management of warfarin. As a result, it can be confirmed that all measures of knowledge were improved. Furthermore, self-management outcomes such as adherence, QOL, efficacy, and satisfaction with usability were more improved than the control group in most studies. Clinical indicators of INR maintenance and mortality and readmission were also effective. However, in Lee et al.’s study, QOL, satisfaction, symptom inventory, and medication adherence, excluding knowledge, were not effective [32].

### 3.4. Assessment of Study Quality

Table 3 presents the quality assessment of the 12 remaining studies. The methodological quality of the 12 studies varied from poor to excellent. The interpretation of the total score is as follows: excellent (26–28), good (20–25), fair (15–19), and poor (≤14). Four studies were classified as having a fair methodological quality. One study had excellent methodological quality, and three studies had a good methodological quality rating. Four studies had poor methodological quality ratings of 14 or less. The most common limitations were related to confounding internal validity. These included randomizing participants to treatment, allocating concealed treatments to investigators and participants, appropriate adjustments for confounding, and consideration of losses during follow-ups. 

## 4. Discussion

This systematic review conducted a detailed analysis of available mobile health applications aimed at controlling anticoagulation and improving self-management programs among adults taking warfarin. The significant variability in the self-management intervention types, their contents, and measured outcomes posed limitations in interpreting and recommending the use of diverse interventions among adults using mHealth applications. Additionally, further research should be kept in mind according to the application’s future development since most of the papers (*n* = 9) included were published within the past five years. 

The systematic review found that there were only a few studies with an unbiased methodological quality of research. Thus, there is a need for larger, more rigorously designed, and randomized controlled trial studies related to educational effectiveness. Moreover, these studies must consider each patient group’s characteristics. The studies included in this systematic review had poor methodological quality. This is mainly due to insufficient power and external validity because they used small samples and only one group pre-post-test design [12,30,31,32]. However, the cohort design’s quality was not high despite the large sample size due to the confounding internal validity and external validity [4,26,27]. A high-quality cohort design was not pursued because participant enrollment and exogenous variables could not be controlled. Furthermore, rather than improving self-management through education, the measurement index focused on clinical indicators such as INR values and complications [4,26,34]. It can be seen that the program was applied using the mHealth application, even though the quality is not high. Moreover, the clinical indicators and self-management indicators were tried as outcomes. Methodologically high-quality studies were randomized controlled trials reported with a median effect size. Here, control and experimental groups were conducted separately. Even a well-planned randomized controlled trial with a small number of patients in a single center is an obstacle [31]. Therefore, improving patient-centered care requires global collaboration to conduct large-scale, multi-centered, and randomized controlled trials with a rigorous design. Since participants’ self-management is evaluated using the mobile health application, repeated measures of knowledge, QOL, self-efficacy, and medication adherence are required as outcomes in future studies.

Oral anticoagulation knowledge, self-efficacy, medication adherence, QOL, and satisfaction were included as self-management outcomes in this systematic review. However, each study had different outcome indicators to check self-management. Even with the same indicator, the assessment tools used were different. As such, there was a limit to comparing the results. Educational content is an important component of self-management applications. It is commonly reported as the most common self-management feature identified within health applications [17,18,36]. Education tailored to each individual is a key recommendation that significantly promotes self-management in the adult population, particularly in terms of medication adherence. Moreover, it likewise promotes self-management’s other aspects by increasing competence and reducing anxiety [37]. Nevertheless, education is not always a priority in mobile health applications [4,27,28,29]. In some studies, self-management through education was not confirmed. Here, only the INR values, observation of complications-related symptoms, and alarms for taking warfarin were confirmed [26,27,28,29]. The comprehensive educational content across mHealth applications for patients taking warfarin was a positive outcome of this review. The majority of the studies confirmed anticoagulant knowledge, efficacy, QOL, and medication adherence. Thus, these showed the mobile application program’s effectiveness. Additionally, it was effective in lowering clinical outcomes such as the readmission, mortality, and complication rate. However, continuous research is needed because the tools (i.e., OAK, MMAS, and PHQ) represented by self-management outcomes are not used consistently. Patient-reported outcomes’ importance is growing. Effective training using mHealth applications for participants needing lifelong management through warfarin control is an intervention consistent with advanced science. This is appropriate even during the COVID-19 pandemic. The development of applications’ contents suitable for the participants’ self-management and the development of application-based health promotion tools for reducing the health inequality gap should be explored through participants with various health literacy levels [18]. Moreover, the mHealth application’s core functionality relies on the use and storage of personally identifiable information. Thus, significant improvements are needed to ensure future comprehensive and transparent data security regulations. 

The number of people taking warfarin for the treatment of cardiovascular diseases (i.e., atrial fibrillation and valve replacement) is increasing worldwide [18,38]. Hence, it is urgent to confirm the contents for self-management by applying the mHealth smartphone application and conducting a rigorous randomized study in multi-centers. Additionally, the results of repeated clinical trials using rigorous methods should be further confirmed in the future since most of the studies were conducted within the last five years. 

This systematic literature review has some limitations. The patients’ reasons for taking warfarin and their underlying medical conditions varied. It is also possible that studies conducted in other languages were excluded, including only studies published in English or Korean. Thus, not all mobile health programs may have been evaluated. The study included in the review had small sample sizes and only one group pre-posttest design and cohort design. Therefore, it was difficult to generalize self-management improvement as the mobile health applications’ effect. The studies included in this review did not report treatment fidelity. This limited their ability to decide the interventions’ true effect on whether biases were potentially involved. It was also limited by homogeneous samples, reducing the results’ generalizability.

## 5. Conclusions

The present systematic literature review confirmed the effects and outcomes of using mHealth applications as a self-management intervention for patients taking warfarin. Various self-management interventions with mHealth applications were included. However, there was a lack of consistency in their impact on clinical and self-management outcomes. Future research may use larger sample sizes and more rigorous randomized controlled trials to obtain a more consistent self-care education program content. Moreover, it is necessary to confirm these applications’ educational effect using the tool represented by the self-management outcomes. Consequently, this systematic literature review reports a lack of commercial self-administered applications with sufficient basis for patient use, given the reasons for warfarin use in hospital settings. Thus, it is necessary to continuously reiterate the customized educational contents and self-management indicators to the target patients. This systematic literature review highlights the need for a comprehensive self-management application that includes clinically verified educational information, medication adherence, and decision-making tools that can be confirmed as outcome indicators. 

## Figures and Tables

**Table 1:** Search strategy.

PubMed	(“Anticoagulants”[Mesh] OR Anticoagulants[ti] OR Anticoagulation[ti] OR “Warfarin”[Mesh] OR Warfarin[ti]) AND (“Mobile health application”[ti] OR Telemedicine[ti] OR “Telemedicine”[mesh] OR “Mobile Health”[ti] OR mHealth[ti] OR “Mobile Applications”[Mesh] OR “Mobile App”[ti] OR “Mobile Applications”[ti] OR “Mobile Application”[ti] OR “home monitoring”[ti] OR Platform[ti] OR “Smartphone”[Mesh] OR “Smart phone”[ti] OR Smartphone[ti] OR “Cell Phone”[ti] OR “Cell Phones”[ti] OR “Mobile Phone”[ti] OR “Mobile Phones”[ti])
Embase	(‘anticoagulant agent’/mj OR ‘anti coagulant’:ti OR ‘anti coagulant agent’:ti OR ‘anti coagulant drug’:ti OR ‘anti coagulating agent’:ti OR … OR ‘wafarin’:ti OR ‘waran’:ti OR ‘warf compound 42’:ti OR ‘warfar’:ti OR ‘warfarin’:ti OR ‘warfarin 2 (dimethylamino) ethanol’:ti OR ‘warfarin potassium’:ti OR ‘warfarin sodium’:ti OR ‘warfarine’:ti OR ‘warfarinum sodium’:ti OR ‘warfil 5’:ti OR ‘warfilone’:ti OR ‘warnerin’:ti ) AND (‘mobile health application’/mj OR ‘moovcare’:ti OR ‘neomate’:ti OR ‘ispo2 (mobile health application)’:ti OR ‘mobile health application’:ti OR … OR ‘smartphone’/mj OR ‘smart phone’:ti OR ‘smartphone’:ti OR ‘smartphones’:ti OR ‘mobile phone’/mj OR ‘cell phone’:ti OR ‘cell phones’:ti OR ‘cellphone’:ti OR ‘cellphones’:ti OR ‘cellular phone’:ti OR ‘cellular telephone’:ti OR ‘mobile phone’:ti OR ‘mobile telephone’:ti)
CINAHL	((MM “Anticoagulants+”) OR (MM “Warfarin”) OR TI (Anticoagulants OR Anticoagulation OR Warfarin)) AND ((MM “Telemedicine+”) OR (MM “Telehealth+”) OR (MM “Mobile Applications”) OR (MM “Cellular Phone+”) OR (MM “Smartphone”) OR TI (“Mobile health application” OR Telemedicine OR “Mobile Health” OR Telehealth OR mHealth OR “Mobile App” OR “Mobile Applications” OR “Mobile Application” OR “home monitoring” OR Platform OR “Smart phone” OR Smartphone OR “Cellular Phone” OR “Cell Phone” OR “Cell Phones” OR “Mobile Phone” OR “Mobile Phones”))
Cochrane	((MeSH descriptor: [Anticoagulants] explode all trees) OR (MeSH descriptor: [Warfarin] explode all trees) OR ((Anticoagulants OR Anticoagulation OR Warfarin):ti)) AND ((MeSH descriptor: [Telemedicine] explode all trees) OR (MeSH descriptor: [Mobile Applications] explode all trees) OR (MeSH descriptor: [Smartphone] explode all trees) OR (MeSH descriptor: [Cell Phone] explode all trees) OR ((“Mobile health application” OR Telemedicine OR “Mobile Health” OR Telehealth OR mHealth OR “Mobile App” OR “Mobile Applications” OR “Mobile Application” OR “home monitoring” OR Platform OR “Smart phone” OR Smartphone OR “Cellular Phone” OR “Cell Phone” OR “Cell Phones” OR “Mobile Phone” OR “Mobile Phones”):ti))

**Table 2:** Characteristics of the mobile health program and detailed contents of the participants taking warfarin.

Author Year Country	Research Design	Sample Size	Age	Setting	Inclusion Criteria	Program	Contents	Study Outcomes	Duration of Intervention	Teaching Methods
Guhl et al. 2020 USA	RCT	120 Exp. 61 Cont. 59	72.1 ± 9.1	Hospital	∙ age ≥ 18 years ∙ history of chronic AF ∙ prescribed oral anticoagulation ∙ English-speaking	Kardia	∙ education ∙ symptoms ∙ adherence ∙ patient activation	∙ HRQoL with the Atrial Fibrillation Effect on Quality of Life (AFEQT) ∙ self-reported adherence	30 days	mHealth app (Kardia apps)
Guo et al. 2017 China	RCT	209 Exp. 113 Cont. 96	69.0	Hospital	∙ age > 18 years ∙ patients with atrial fibrillation ∙ patients without valvular atrial fibrillation	Mobile AF (mAF)	∙ clinical decision-support tools (CHA_2_DS_2_-VASc, HAS-BLED, SAMe-TT_2_R_2_) ∙ education program ∙ self-care items (Euro EQ-5D-Y) ∙ structured follow-up	∙ knowledge ∙ QOL ∙ drug adherence ∙ anticoagulation satisfaction ∙ usability, feasibility, acceptability of the mAF App	3 months	mHealth app
Guo et al. 2020 China	RCT	3324 Exp. 1646 Cont. 1678	68.5	Hospital	∙ age ≥ 18 years ∙ patients with a new-onset of paroxysmal, persistent, or permanent atrial fibrillation with ECG ∙ CHA_2_DS_2_-VASc ≥ 2	mAFA	∙ clinical decision-support tools (CHA_2_DS_2_-VASc, HAS-BLED, SAMe-TT_2_R_2_) ∙ education program ∙ self-care items (Euro EQ-5D-Y) ∙ structured follow-up ∙ the ABC pathway for AF management	∙ clinical outcomes (thromboembolism, bleeding events, cardiovascular outcomes, all-cause death, re-hospitalization, composite outcome)	12 months	mHealth app
Guo et al. 2020 China	RCT	2473 Exp. 1261 Cont. 1212	56.7 ± 13.7	Hospital	∙ age > 18 years ∙ patients with atrial fibrillation with ECG ∙ CHA_2_DS_2_-VASc ≥ 2	mAFAII	∙ the ABC pathway for AF management	∙ clinical outcomes (thromboembolism, bleeding events, recurrent AF or AF symptom, heart failure, re-hospitalization. all-cause death) ∙ adherence and persistence	1 year	mHealth app
Labovitz et al. 2017 USA	RCT	28 Exp. 15 Cont. 13	57.0 ± 13.2	Hospital	∙ patients diagnosed with ischemic stroke ∙ oral anticoagulation therapy		∙ visual confirmation of ingestion ∙ self-reported dose via the AI app ∙ self-reported dose by clinic staff ∙ missed dose ∙ dose taken in a clinic	∙ adherence ∙ PT/INR, APTT ∙ usability and feasibility	12 weeks	AI platform
Lee et al. 2016 USA	One group pre-posttest design	21	67.3 ± 8.7	Hospital	∙ age ≥ 55 years ∙ taking an oral anticoagulant ∙ English-speaking or Spanish-speaking ∙ not cognitively impaired ∙ not living in a long-term care facility	Mobile Applications for Seniors to enhance Safe anticoagulation therapy (MASS)	∙ education about anticoagulation therapies and safety tips ∙ medication self-monitoring and reminders ∙ Vitamin K content of foods ∙ monitoring signs and symptoms of bleeding ∙ monitoring blood (INR) ∙ connecting with trusted other people ∙ message to tell doctors	∙ OAK test ∙ Perception of Anticoagulant Treatment Questionnaire (PACTQ) ∙ Duke Anticoagulation Satisfaction Scale (DASS) ∙ Patient Health Questionnaire (PHQ-9) ∙ Brief Symptom Inventory (BSI) ∙ Morisky medication adherence scale (MMAS) ∙ Perceived Health Web Site Usability Questionnaire (PHWSUQ-12)	3 months	mHealth app
Lin et al. 2014 Taiwan	Retrospective cohort design	7278 Exp. 3781 Cont. 3497	59.3	Hospital	∙ patients engaged in warfarin therapy	Personal Handy-phone System (PHS)	∙ PHS alert system	∙ PT/INR ∙ complications (thromboembolic or hemorrhagic events) ∙ major hemorrhage, event ∙ treatment with vitamin K	2 years	Mobile phone network system
Prochaska et al. 2017 Germany	Prospective cohort design	2318 Exp. 760 Cont. 1558	73.0	Hospital	∙ patients with an oral anticoagulation therapy	Poltavita	∙ INR values ∙ computer-assisted dosing algorithms	∙ efficacy ∙ safety ∙ all-cause mortality ∙ hospitalization	12–14 months	web-based electronic patient file
Prochaska et al. 2015 Germany	Prospective cohort design	2771 Exp. 760 Cont. 2011	73.0	Hospital	∙ age ≥ 18 years ∙ taking an oral anticoagulant ≥ 4 months	ThrombEVAL study	∙ self-management of OAC with INR values	∙ TTR of INR	3 months	Telemedicine-based coagulation service
Shilbayeh et al. 2019 Saudi Arabia	One group pre-posttest design	45	45.8 ± 12.8	Hospital	∙ age > 18 years ∙ Arabic-speaking ∙ receiving OAT (mainly warfarin) and visiting the clinic regularly ∙ a naïve patient ∙ using smartphones or tablets with Android	Coagulation and Anticoagulant Therapy and Awareness (CATA)	∙ Oral anticoagulation knowledge (OAK) scale ∙ Anti-clot Treatment Scale (ACTS) ∙ Treatment Satisfaction Questionnaire for Medication (TSQM 1.4)	∙ Oral anticoagulation knowledge (OAK) scale ∙ Anti-clot Treatment Scale (ACTS) ∙ Treatment Satisfaction Questionnaire for Medication (TSQM 1.4)	45 days	mHealth app
Stephan et al. 2018 Brazil	One group pre-posttest design	30 Exp. 20 Pilot 10	67.7 ± 9.4	Hospital	∙ patients with AF and low socioeconomic and cultural status ∙ using OAC		∙ knowing the disease (video) ∙ individualizing the risks ∙ understanding risks and benefits ∙ knowing the treatment option ∙ making a choice	∙ AF knowledge questionnaire ∙ Decisional Conflict Scaled in Health (DSCH)	During medical visits	dactor’s tablet computer
Talboom-Kamp et al. 2017 Netherlands	Parallel cohort design	195 E-learning 52 Group course 58 Basic training 85	66.9	Hospital	∙ long-term indication for anticoagulants ∙ self-management	PORTALS study: Poltavita	∙ disease-specific knowledge ∙ self-testing skills ∙ use of the web portal ∙ self-adjustment of medication	∙ TTR of INR ∙ usage of an eHealth platform ∙ Generalized Self-Efficacy Scale (GSES), education level	18 months	eHealth platform

Abbreviations: RCT = randomized controlled trial; PT/INR = prothrombin time international normalized ratio; APTT = activated partial thromboplastin time; HRQoL = health-related quality of life; TTR = time in therapeutic range; QOL = quality of life; EQ-5D-Y = EuroQoL 5-dimensions youth version; AF = atrial fibrillation; CHA_2_DS_2_-VASc = congestive heart failure, hypertension, age ≥ 75 years, diabetes mellitus, stroke/transient ischemic attack, vascular disease, age 65 to 74 years, sex category; HAS-BLED = hypertension, abnormal renal/liver function, stroke, bleeding history or predisposition, labile international normalized ratio, elderly (>65 years), drugs/alcohol concomitantly; SAMe-TT_2_R_2_ = sex, race, medical history, tobacco use, race; ABC pathway = anticoagulation to avoid stroke, better symptom management, cardiovascular risk and comorbidity management; AI = artificial intelligence.

**Table 3:** Quality assessment.

Author (Year)	Reporting (11) *	External Validity (3) *	Internal Validity: Bias (7) *	Internal Validity: Confounding (6) *	Power (1) *	Total Score (28)
Guhl et al. (2020)	9	3	7	4	1	24
Guo et al. (2017)	10	3	7	5	1	26
Guo et al. (2020)	10	3	6	4	1	24
Guo et al. (2020)	10	3	6	5	1	24
Labovitz et al. (2017)	6	0	3	2	0	11
Lee et al. (2016)	7	0	5	3	0	15
Lin et al. (2014)	7	1	4	2	1	15
Prochaska et al. (2015)	6	1	5	2	1	15
Prochaska et al. (2017)	6	1	4	2	1	14
Shilbayeh et al. (2019)	7	0	5	2	0	14
Stephan et al. (2018)	6	0	5	2	0	13
Talboom-Kamp et al. (2017)	8	1	5	2	0	16

* maximum score in each subscale.
